# Supplementary material for: Implementing a universal gate set on a logical qubit encoded in an oscillator
Source: Nat Commun. 2017 Jul 21;8:94. doi: 10.1038/s41467-017-00045-1 (PMC5522494; doi:10.1038/s41467-017-00045-1)
Supplement: Supplementary file 2 — Supplementary Information [file 41467_2017_45_MOESM2_ESM.pdf]

SI GUIDE

File Name: Supplementary Information
Description: Supplementary Figures, Supplementary Notes and Supplementary References.

File Name: Peer Review File
Description:

Supplementary Note 1. SYSTEM HAMILTONIAN

Here we give the full system Hamiltonian to the precision with which we have characterized it. We denote the annihilation operator corresponding to the oscillator (transmon) mode with $\hat{a}$ ($\hat{b}$). Breaking down the system Hamiltonian into components representing the individual modes, their interactions, as well as driving terms, we can write

$$H(t) = H_{\text{oscillator}} + H_{\text{transmon}} + H_{\text{interaction}} + H_{\text{drive}}(t) \quad (1)$$

$$H_{\text{oscillator}}/\hbar = \omega_{\text{C}}\hat{a}^{\dagger}\hat{a} + \frac{K}{2}(\hat{a}^{\dagger})^2\hat{a}^2 \quad (2)$$

$$H_{\text{transmon}}/\hbar = \omega_{\text{T}}\hat{b}^{\dagger}\hat{b} + \frac{\alpha}{2}(\hat{b}^{\dagger})^2\hat{b}^2 \quad (3)$$

$$H_{\text{interaction}}/\hbar = \chi\hat{a}^{\dagger}\hat{a}\hat{b}^{\dagger}\hat{b} + \frac{\chi'}{2}\hat{b}^{\dagger}\hat{b}(\hat{a}^{\dagger})^2\hat{a}^2 \quad (4)$$

$$H_{\text{drive}}(t)/\hbar = \epsilon_{\text{C}}(t)\hat{a} + \epsilon_{\text{T}}(t)\hat{b} + \text{h.c.} \quad (5)$$

When simulating how known decoherence sources should impact the fidelity of our operations, we use a Markovian Lindblad master equation of the form:

$$\frac{\partial}{\partial t}\rho(t) = \frac{-i}{\hbar}[H(t),\rho(t)] + \left(\frac{1}{T_{1,\text{C}}}D[\hat{a}] + \frac{1}{T_{1,\text{T}}}D[\hat{b}] + \frac{1}{T_{\phi}}D[\hat{b}^{\dagger}\hat{b}]\right)(\rho(t)) \quad (6)$$

$$D[a](\rho) = a\rho a^{\dagger} - \frac{1}{2}\{a^{\dagger}a,\rho\} \quad (7)$$

The measured values for each of these system parameters are shown in Supplementary Table 1.

System Parameter	Hamiltonian Term	Parameter Value
Transmon frequency	$\omega_{\text{T}}\hat{b}^{\dagger}\hat{b}$	$2\pi \times 5664.0$ MHz
Oscillator frequency	$\omega_{\text{C}}\hat{a}^{\dagger}\hat{a}$	$2\pi \times 4452.6$ MHz
Dispersive shift	$\chi\hat{a}^{\dagger}\hat{a}\hat{b}^{\dagger}\hat{b}$	$2\pi \times -2194 \pm 3$ kHz
Transmon anharmonicity	$\frac{\alpha}{2}(\hat{b}^{\dagger})^2\hat{b}^2$	$2\pi \times -236$ MHz
Oscillator anharmonicity (Kerr)	$\frac{K}{2}(\hat{a}^{\dagger})^2\hat{a}^2$	$2\pi \times -3.7 \pm 0.1$ kHz
Second order dispersive shift	$\frac{\chi'}{2}(\hat{a}^{\dagger})^2\hat{a}^2\hat{b}^{\dagger}\hat{b}$	$2\pi \times -19.0 \pm 0.4$ kHz
Transmon relaxation	$\frac{1}{T_1}D[\hat{b}]$	$170 \pm 10\mu s$
Transmon dephasing	$\frac{1}{T_{\phi}}D[\hat{b}^{\dagger}\hat{b}]$	$43 \pm 5\mu s$
Oscillator relaxation	$\frac{1}{T_{\text{cav}}}D[\hat{a}]$	$2.7 \pm 0.1ms$

Supplementary Table 1: **Measured system parameters** The dispersive shift and its second order correction are determined using transmon spectroscopy experiments (Supplementary Figure 1). The oscillator anharmonicity is determined by fitting a set of Wigner functions after different lengths of free evolution time.

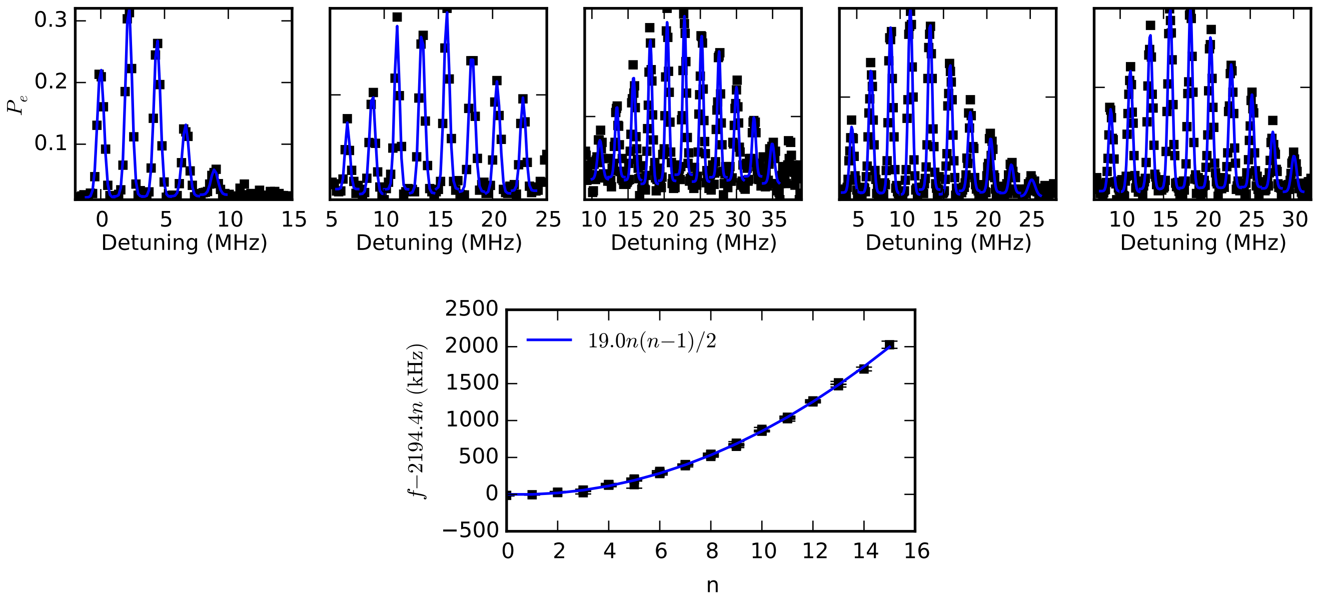

Supplementary Figure 1: **Dispersive shift measurement.** The dispersive shift χ and its second order correction term χ' are determined from transmon spectroscopy experiments with several different displacements (top). Each peak is fit to a Gaussian and the resulting center frequencies are fit using a quadratic model.

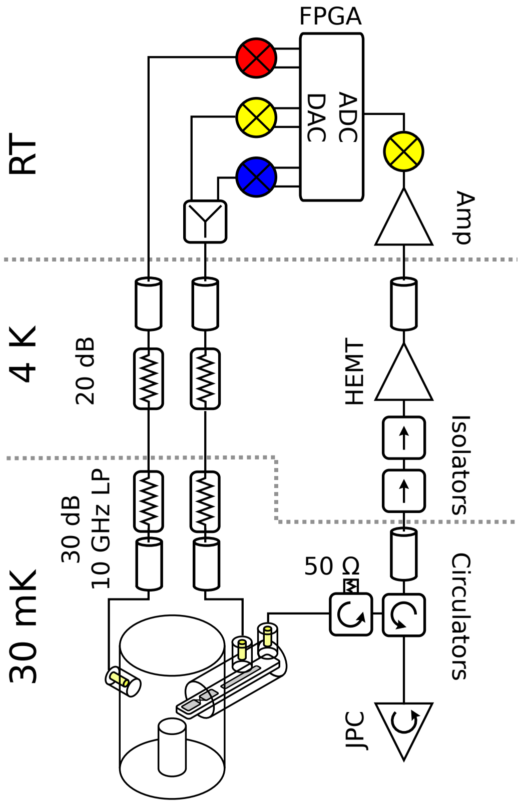

Supplementary Figure 2: **Measurement setup.** An FPGA controller (2x Innovative Integration X6-1000M in VPXI-ePC chassis) generates 3 pairs of I/Q waveforms using 500 Msample/s digital to analog converters (DAC). Each pair is upconverted using an I/Q mixer (Marki IQ-0307-LXP or IQ-0618-LXP depending on the frequency). The color of the mixer indicates the local oscillator: red for the storage, yellow for the readout and blue for the transmon. To prevent problems due to mixer leakage, each local oscillator is set 50 MHz above the desired frequency and single-sideband modulation is used. Proper attenuation at each temperature stage is crucial to thermalize the black-body radiation from the 50Ω environment. Additional low-pass filters (K&L250-10000 and home-built eccosorb) protect the sample from spurious high-frequency components. The output chain consists of a Josephson Parametric Converter (JPC), which reflects the input signal with ~ 20 dB of gain (bandwidth ~ 6 MHz). The circulators (Pamtech XTE0812KC) prevent the amplified signal from going back to the sample and direct it through 2 isolators (Pamtech CWJ0312KI) to a HEMT-amplifier (Low Noise Factory LNF-LNR1_12A). Finally, an image reject mixer (Marki SSB-0618) converts the RF signal back to the intermediate frequency (50 MHz). The FPGA samples the signal using a 1 Gsample/s analog to digital converter (ADC), demodulates and integrates to give one bit of information indicating whether the transmon was in $|g\rangle$ or $|e\rangle$.

Supplementary Note 2. GRAPE IMPLEMENTATION

We define operations on our system in terms of a set of simultaneous state transfers, i.e. the operation should, for each i, take the initial state $\left|\psi^{(i)}_{\text{init}}\right\rangle$ to the corresponding final state $\left|\psi^{(i)}_{\text{final}}\right\rangle$. In order to prepare a desired operation on the joint oscillator-transmon Hilbert space, we use GRAPE to maximize the (coherent) average fidelity of these state transfers over the controls $\epsilon(t) \equiv (\epsilon_{\text{C}}(t), \epsilon_{\text{T}}(t))$:

$$\underset{\epsilon(t)}{\text{maximize}} \;\; \mathcal{F}\left(\epsilon(t)\right) \tag{8}$$

$$\mathcal{F}\left(\epsilon(t)\right) = \left|\sum_i \left\langle \psi^{(i)}_{\text{final}} \middle| U\left(T, \epsilon(t)\right) \middle| \psi^{(i)}_{\text{init}} \right\rangle\right|^2, \tag{9}$$

where the unitary U defined by the waveforms $\epsilon(t)$ is given by the time-ordered exponential of the Hamiltonian up to some final time T,

$$U(T, \epsilon(t)) = \mathcal{T} \exp\left(-\int_0^T \mathrm{d}t\, H\left(\epsilon(t)\right)\right). \tag{10}$$

To make the problem numerically tractable, $\epsilon(t)$ is represented as a piecewise constant function with $N = T/\Delta t$ steps of length $\Delta t = 2$ ns, corresponding to the time resolution of our arbitrary waveform generator.

$$U\left(T, \epsilon(t)\right) = U_N U_{N-1} \cdots U_2 U_1 \tag{11}$$

$$U_k = \exp\left(\frac{i\Delta t}{\hbar} H(\epsilon(k\Delta t)\right) \tag{12}$$

Using 4 parameters per time point (real and imaginary components of the oscillator and transmon drives) and $N = 550$ time points representing the 1.1 μs pulse, there are 2200 parameters to optimize over. In order to carry out a numerical optimization with such a large number of parameters, it is crucial that one can efficiently calculate the gradient of the optimized function with respect to all of its parameters. In this case it is possible to use Quasi-Newton optimization algorithms, such as L-BFGS [1] in order to optimize the function with many fewer function evaluations. We can simplify the calculation of the gradient as follows:

$$\frac{\partial \mathcal{F}}{\partial \epsilon_i(k\Delta t)} = 2\left(\text{Re}(v)\text{Re}\left(\frac{\partial v}{\partial \epsilon_i(k\Delta t)}\right) + \text{Im}(v)\text{Im}\left(\frac{\partial v}{\partial \epsilon_i(k\Delta t)}\right)\right) \tag{13}$$

$$v \equiv \sum_i \left\langle \psi^{(i)}_{\text{final}} \middle| U\left(T, \epsilon(t)\right) \middle| \psi^{(i)}_{\text{init}} \right\rangle \tag{14}$$

$$\frac{\partial v}{\partial \epsilon_i(k\Delta t)} = \sum_i \left\langle \psi^{(i)}_{\text{final}} \middle| \frac{\partial U\left(T, \epsilon(t)\right)}{\partial \epsilon_i(k\Delta t)} \middle| \psi^{(i)}_{\text{init}} \right\rangle \tag{15}$$

$$= \sum_i \left\langle \psi^{(i)}_{\text{final}} \middle| U_N \cdots U_{k+1} \frac{\partial U_k}{\partial \epsilon_i(k\Delta t)} U_{k-1} \cdots U_1 \middle| \psi^{(i)}_{\text{init}} \right\rangle \tag{16}$$

Therefore, the calculation of the gradient can be reduced to computing the states $U_{k-1} \cdots U_1 \left|\psi^{(i)}_{\text{init}}\right\rangle$, $U^\dagger_{k+1} \cdots U^\dagger_N \left|\psi^{(i)}_{\text{final}}\right\rangle$ as well as the gradient of the step propagator $\frac{\partial U_k}{\partial \epsilon_i(k\Delta t)}$. The states can be stored from the evaluation of the fidelity itself, and there are several efficient ways of evaluating the gradient of the propagator [2].

The optimization problem defined by equation 8 is generally underdetermined, i.e. there are many solutions $\epsilon(t)$ which achieve equally high fidelities. Therefore, we can add additional terms to the optimization cost function, such that the resulting solution optimizes against several other desiderata. For a set of constraints on the solution $\{g_i \geq 0\}$, where ideally $g_i\left(\epsilon(t)\right) = 0$, we can associate a Lagrange multiplier λ_i, and modify our optimization to read:

$$\underset{\epsilon(t)}{\text{maximize}} \;\; \mathcal{F}\left(\epsilon(t)\right) - \sum_i \lambda_i g_i\left(\epsilon(t)\right) \tag{17}$$

The values λ_i are chosen by trial-and-error, set to be just large enough that the violation of the constraint upon termination is within acceptable levels. For instance, since the output power of our AWG is limited, the pulse must obey $\epsilon(t) \leq \epsilon_{\text{max}}$ for all t. We can construct a penalty term of the form

$$g_{\text{amplitude}}\left(\epsilon(t)\right) = \int \mathrm{d}t \left(|\epsilon(t)| - \epsilon_{\text{max}}\right)^2 \Theta\left(|\epsilon(t)| - \epsilon_{\text{max}}\right) \tag{18}$$

$$= \sum_n \left(|\epsilon(n\Delta t)| - \epsilon_{\text{max}}\right)^2 \Theta\left(|\epsilon(n\Delta t)| - \epsilon_{\text{max}}\right) \tag{19}$$

Since the transfer function of the lines between the AWG and the experimental system becomes more and more uncertain as one moves further away from resonance, it is also desirable to minimize the bandwidth of the applied pulses, we do this in two ways. First, we create a penalty term of the form

$$g_{\text{derivative}}\left(\epsilon(t)\right) = \int \mathrm{d}t \left(\frac{\partial \epsilon(t)}{\partial t}\right)^2 \tag{20}$$

$$\rightarrow \sum_n \left(\epsilon((n+1)\Delta t) - \epsilon(n\Delta t)\right)^2 , \tag{21}$$

where equation 21 is the appropriate equivalent of equation 20 for a piecewise constant function. Additionally, we enforce a hard cutoff on the minimum and maximum frequencies allowed in the solution by reparametrizing the optimization problem in terms of the Fourier transform of the pulses [3]:

$$\underset{\tilde{\epsilon}(\omega)}{\text{maximize}} \;\; \mathcal{F}\left(\epsilon(t)\right) - \sum_i \lambda_i g_i\left(\epsilon(t)\right) \tag{22}$$

$$\text{such that} \;\; \tilde{\epsilon}(\omega) = 0 \text{ when } \omega < \omega_{\text{min}} \text{ or } \omega > \omega_{\text{max}}$$

Since computer memory is finite, we are forced to choose a photon number truncation N such that the operator $\hat{a}$ becomes a $N \times N$ matrix. When we do this, we are in effect replacing our infinite-dimensional oscillator with a finite-dimensional qudit. This replacement is only valid if all of the system dynamics relevant for the desired state transfers occurs within the $\{|0\rangle, \ldots, |N-1\rangle\}$ subspace. For generic applied drives this is not the case. In order to enforce this property, we modify the optimization problem to find a solution which operates identically under several different values of N. Writing the fidelity as computed with a truncation N as $\mathcal{F}_N$, we have:

$$\underset{\tilde{\epsilon}(\omega)}{\text{maximize}} \;\; \left(\sum_k \mathcal{F}_{N+k}\left(\epsilon(t)\right)\right) - \left(\sum_i \lambda_i g_i\left(\epsilon(t)\right)\right) \tag{23}$$

To enforce that the behavior is identical in the different truncations, we add the penalty term

$$g_{\text{discrepancy}}\left(\epsilon(t)\right) = \sum_{k_1 \neq k_2} \left(\mathcal{F}_{N+k_1}\left(\epsilon(t)\right) - \mathcal{F}_{N+k_2}\left(\epsilon(t)\right)\right)^2 \tag{24}$$

The choice of N determines the maximum photon number population which can be populated during the pulse, and figures in determining the minimum time necessary for the operation (faster pulses can be achieved with higher N).

Supplementary Note 3. SYSTEM PREPARATION

The system is initialized by cooling of both the storage resonator (typical steady-state population $\sim 2\%$) and the transmon (steady-state population $\sim 5\%$) using measurement-based feedback. The protocol is detailed in Supplementary Figure 3. It proceeds by first establishing that the oscillator is in its ground state, and finishes by ensuring that the transmon is in its ground state (Supplementary Figure 3a). If it is determined that the oscillator is not empty, a set of "Q-Switching" drives is applied which effectively couples the storage mode to the short-lived readout mode (Supplementary Figure 3b). The drives consist of strong tones applied at $\omega_C + \Delta$ and $\omega_{\text{RO}} + \Delta$ with $\Delta = 40$ MHz. The effectiveness of this strategy can be seen from

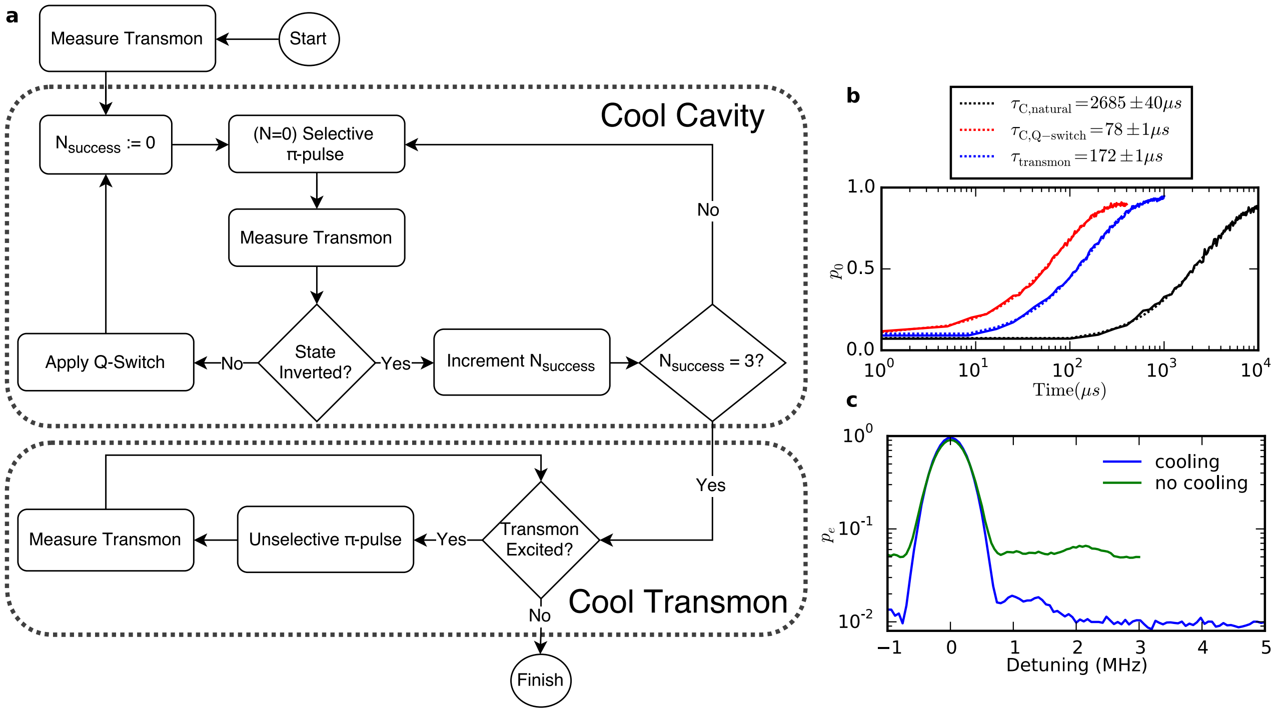

Supplementary Figure 3: **System preparation. a,** System preparation protocol to cool the oscillator as well as the transmon. **b,** Lifetime of the transmon and oscillator. For the oscillator we prepare the Fock state $|1\rangle$ using an optimal control pulse and show a natural decay curve as well as one with Q-switching pumps applied. **c,** Transmon spectroscopy data after system preparation. The "cooling" ("no-cooling") curve are with (without) the feedback-cooling protocol. Photons in the storage (readout) oscillator show up as a peak around $\chi_s \approx 2$ MHz ($\chi_r \approx 1$ MHz).

the transmon spectroscopy traces (Supplementary Figure 3c). The transmon population is reduced to $\sim 1\%$ and the storage resonator population is $\ll 1\%$. A residual population of the readout resonator of about 1% is visible as a peak around 1 MHz detuning. Additionally, this cooling protocol allows for a dramatically increased experimental repetition rate, decreasing the inter-experimental delay τ from $\tau \approx 18$ ms to $\tau < 1$ ms.

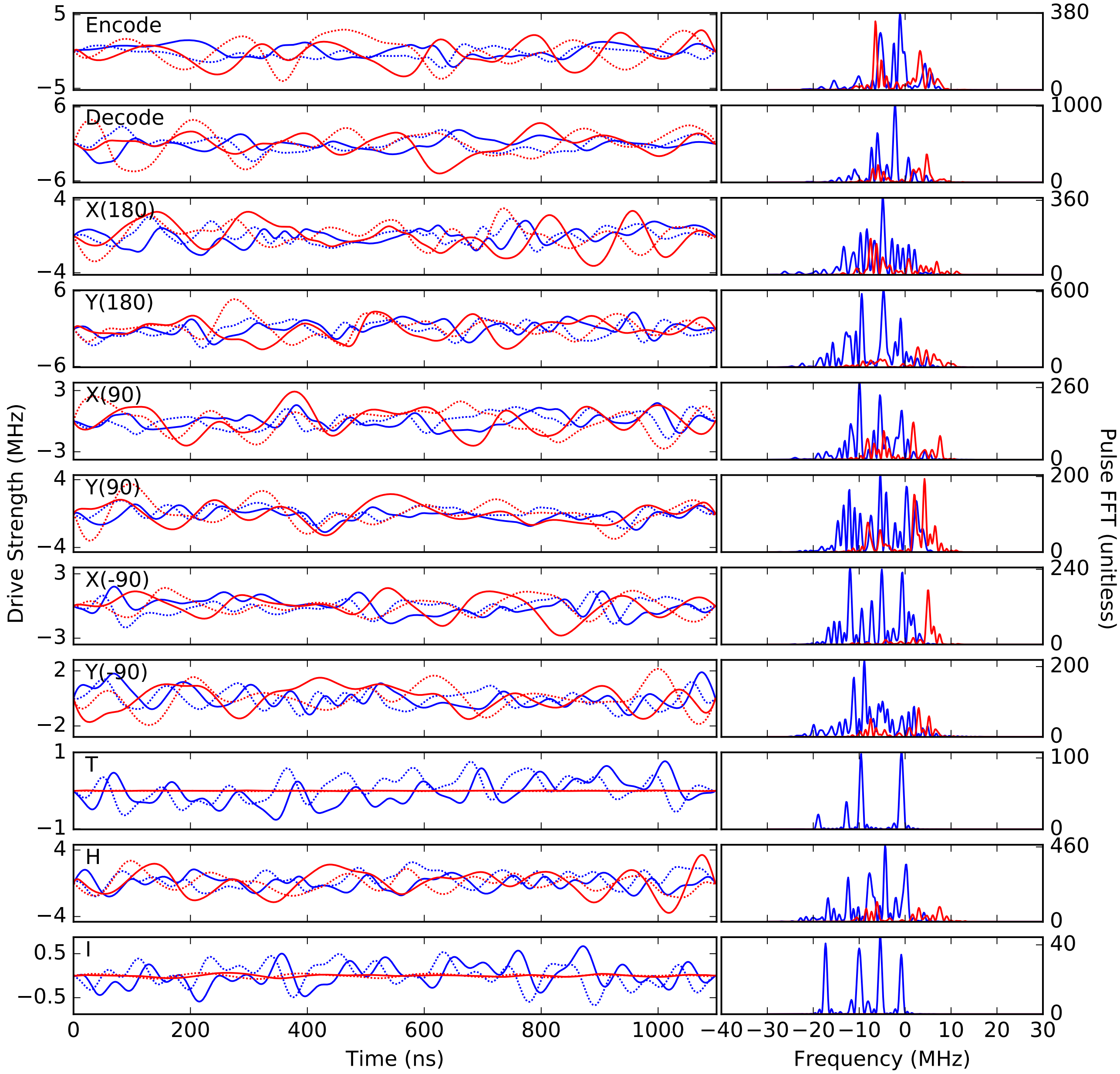

Supplementary Figure 4: **Optimized pulse waveforms.** In the first column, we plot the complex waveforms $\epsilon_{\mathrm{T}}(t)$ and $\epsilon_{\mathrm{C}}(t)$. In the second column, we show the Fourier spectrum $|\tilde{\epsilon}(\omega)|^2$. Blue (red) lines correspond to drives on the transmon (oscillator). Solid (dotted) lines correspond to the in-phase (quadrature) component of the drive. Note that the I and the T gate do not have to change the photon number distribution, but only have to apply different phases to each Fock state component. This can be done by manipulating the transmon [4] only; GRAPE finds a solution with a very small oscillator drive amplitude as well.

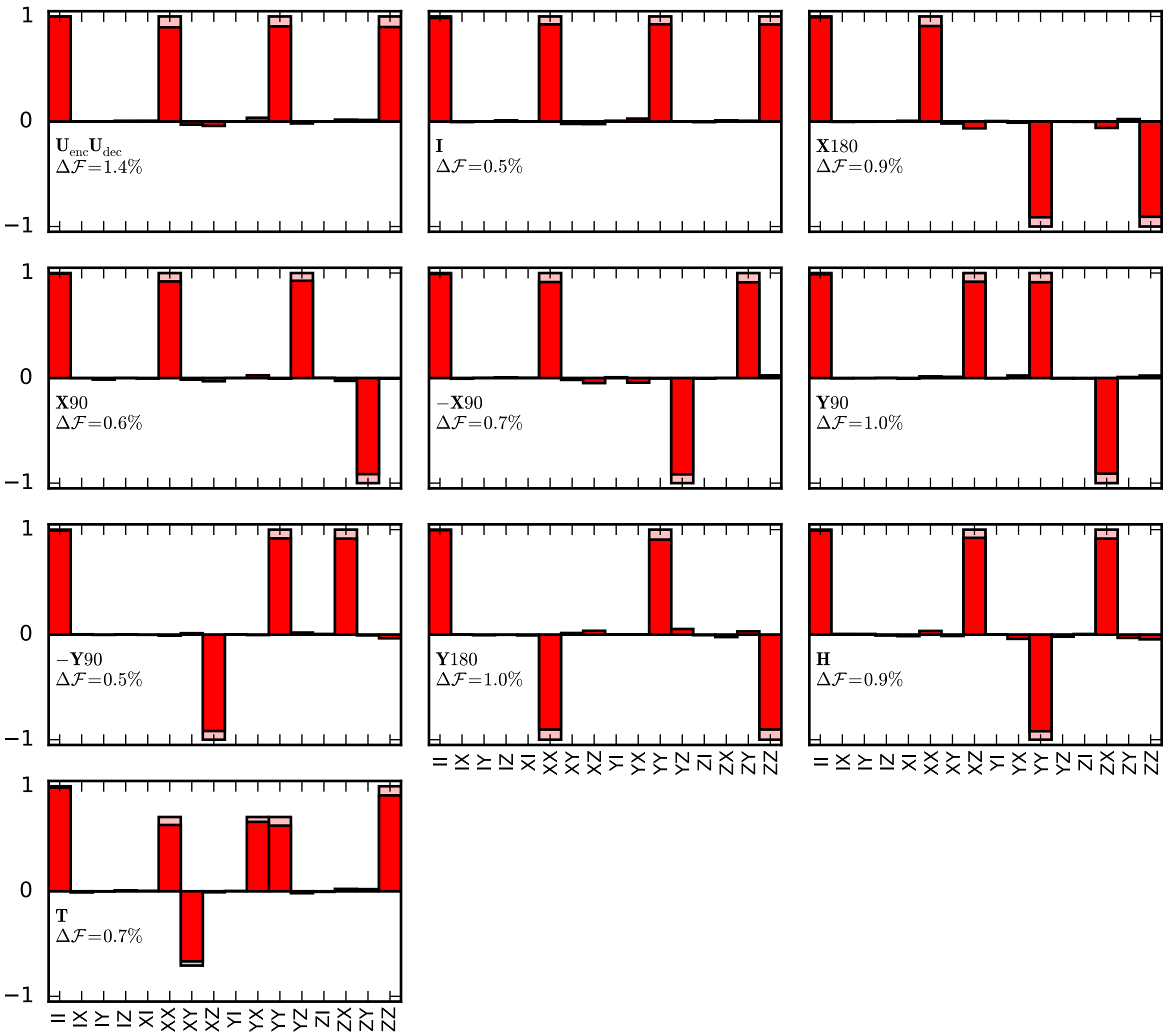

Supplementary Figure 5: **Full Process Tomography Results.** The experimentally determined Pauli bars in dark red, the ideal in light red. The reported $\Delta\mathcal{F}$ is the difference between $\mathcal{F}_{\mathrm{PT}}(\mathrm{U_{dec}U_XU_{enc}})$ and $\mathcal{F}_{\mathrm{PT}}(\mathrm{U_{dec}U_{enc}}) = 0.964$.

Supplementary Note 4. RANDOMIZED BENCHMARKING VALIDITY

The rigorous mathematical validity of randomized benchmarking as a tool to measure average gate fidelity requires that the errors be of a certain type. They must be time-independent, Markovian and gate-independent. If one or more of these properties are violated, the randomized benchmarking results must be analyzed with care. In our case, the vast majority of errors occuring during the operation take states from within to outside the encoded subspace (see Supplementary Table 1). This constitutes a form of leakage error which can cause non-Markovian effects [5]. There are many adjusted RB protocols which attempt to deal with this issue [6–9]. These protocols generally assume the ability to either measure or manipulate states outside of the logical space. Since we did not have easy access to these tools, we instead have performed an unmodified RB protocol and have adjusted the interpretation to account for the possibility of misleading results.

To analyze the potential error, we have simulated randomized benchmarking on a model system which allows for leakage. We consider a two-level system being benchmarked, in addition to a d-dimensional auxilary system. The following protocol is simulated:

Here the U_i are perfect single qubit clifford operations. The Λ_i are the associated error terms which couple the logical and ancilla spaces. These are created via small a random Liouvillian:

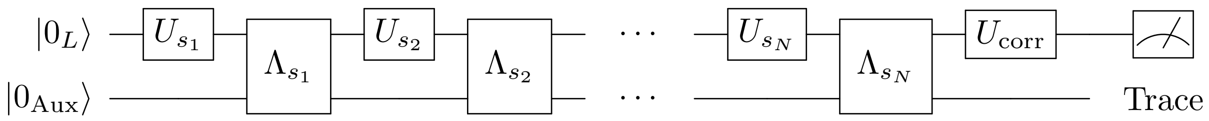

$$\Lambda_i = e^{\epsilon L_i}$$

$$L_i = q[H_i, \cdot] + (1-q) \sum_{k=1}^{4d^2-1} D[A_{i,k}]$$

The A_i and H_i are random $2d \times 2d$ matrices. q scales the relative coherence of the errors, and ϵ is set to adjust the total error level. The naive RB fidelity is calculated by scanning the sequence length N, sampling many random sequences s and fitting the exponential decay. This value can be compared with the underlying average fidelity

$$F_{i,\text{av}} = \int d\psi_L \langle \psi_L, 0 | \Lambda_i \left(|\psi_L, 0\rangle\langle\psi_L, 0| \right) |\psi_L, 0\rangle$$

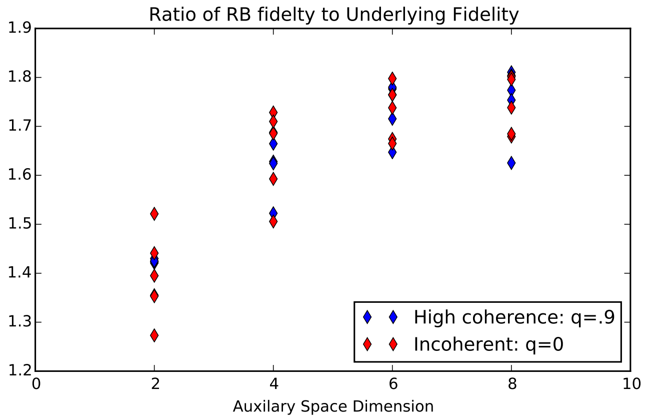

Supplementary Figure 6: **Effect of leakage dimension on randomized benchmarking accuracy**. As a function of the size of the Hilbert space that leakage can occur to, the fidelity as estimated by an RB experiment overestimates the underlying fidelity by a certain factor, which depends sub-linearly on the size of the additional Hilbert space. In our experiment, the size of the Hilbert space is of order 10 and we estimate that a correction factor of 1.7 ± 0.1 is required.

As we see in Supplementary Figure 6, across a range of underlying fidelities (here between .8 and .995), and for both coherent and incoherent errors, a relatively fixed ratio of RB fidelity to underlying fidelity, which does depend, sublinearly, on the dimension of the auxilary space. The RB estimate therefore overestimates the fidelity, but by a small, fixed amount. While we cannot precisely quantify the dimension of the space our errors leak into, we can estimate that the error rate is less than twice that of the naive estimate. This imprecision contributes the largest component to the error in our reporting of the gate fidelity.

Supplementary Note 5. EMPIRICAL TUNING

We use the randomized benchmarking protocol [10, 11] to perform fine tuning of the resulting pulse waveforms. Since the cables and frequency modulation setup between our waveform generator and our device are not spectrally flat, we attempt

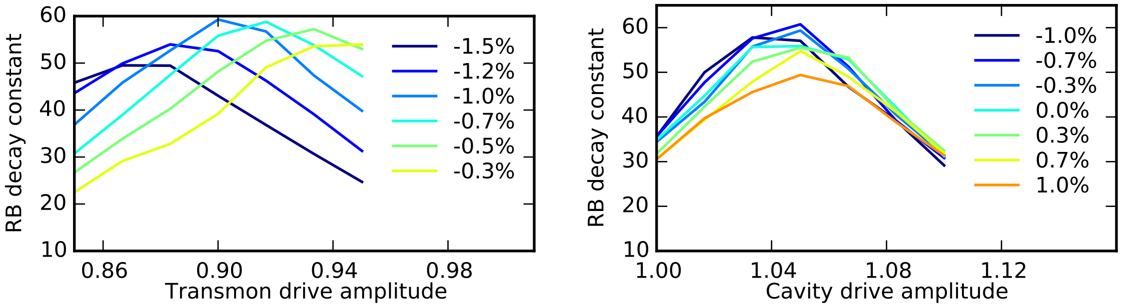

Supplementary Figure 7: **Dispersion and amplitude optimization** The randomized benchmarking decay constant versus transmon drive amplitude for several different dispersion values (in % per MHz). Because of the spectral content of the pulses, the amplitude might have to be corrected when the dispersion value is adjusted.

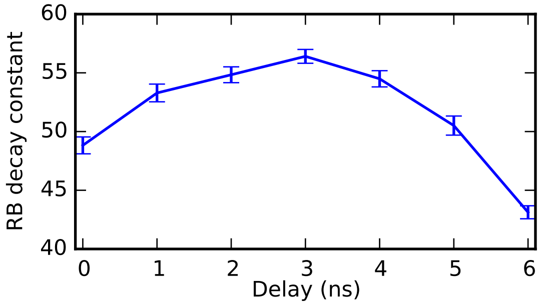

Supplementary Figure 8: **Delay optimization.** RB decay constant as a function of delay time between the transmon and oscillator control fields.

to find a correction to the pulse by applying a linear amplitude weighting in the frequency domain, i.e. Fourier transforming the waves to find $\tilde{\epsilon}(\omega)$, transforming using the weighting coefficient b and delay parameter τ. $\tilde{\epsilon}(\omega) \to (1 + b\omega e^{i\omega\tau})\tilde{\epsilon}(\omega)$, and inverse Fourier transforming to find the corrected waves in the time domain. We can empirically optimize the value of b (Supplementary Figure 7) and τ (Supplementary Figure 8) using randomized benchmarking.

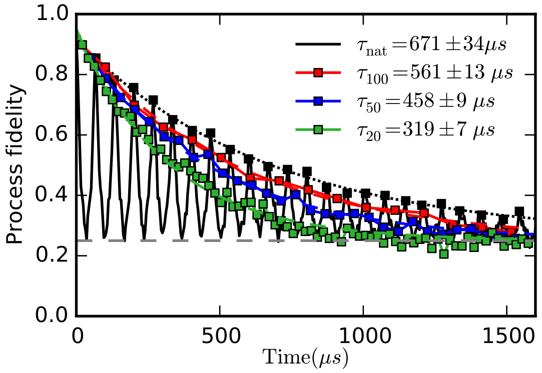

Supplementary Figure 9: **Lifetime of the cat-qubit**. The long-lived resonator is not perfectly harmonic; its Kerr is -3.7 kHz. This nonlinearity will scramble the basis-states of our encoding under free evolution. The black curve shows the process fidelity versus waiting time: periodic revivals [12] associated with rephasing of the basis-states are clearly visible. Note that the revival periodicity is not $1/K$ due to an intentional frequency detuning. Our control pulses take this effect into account during their operating time. Additionally, we can design a control pulse that corrects for the evolution associated with some time Δt of free evolution. The different curves in this plot are the result of a stroboscopic Kerr-correction experiment for 20, 50 and 100 μs of free-evolution. We can infer a Kerr-correction gate error of $\approx 1.7\%$ per gate by extracting the additional decay rate compared to the natural decay.

Gate	P(error)	$P(\lvert e\rangle \mid$ error)	P(detectable | error)
I	0.004	0.480	0.978
X90	0.012	0.517	0.946
-X90	0.013	0.588	0.980
X180	0.016	0.496	0.974
Y90	0.012	0.541	0.965
-Y90	0.012	0.615	0.965
Y180	0.022	0.411	0.981
H	0.015	0.507	0.968
Average	0.013	0.519	0.970
$U_{enc}U_{dec}$	0.027	0.414	0.813
T	0.007	0.495	0.945

Supplementary Table 2: **Error probability and detectability.** Results from master equation simulation of pulses using parameters from Supplementary Table 1. The first column indicates the probability of an error (i.e. ending the pulse in an incorrect state). The second column indicates the probability of finding the transmon in the excited state conditioned on an error having occurred. The third column indicates the probability of ending in a state outside the logical subspace given that an error has occurred, and is the maximum fraction of the errors which could be in principle detected in some way.

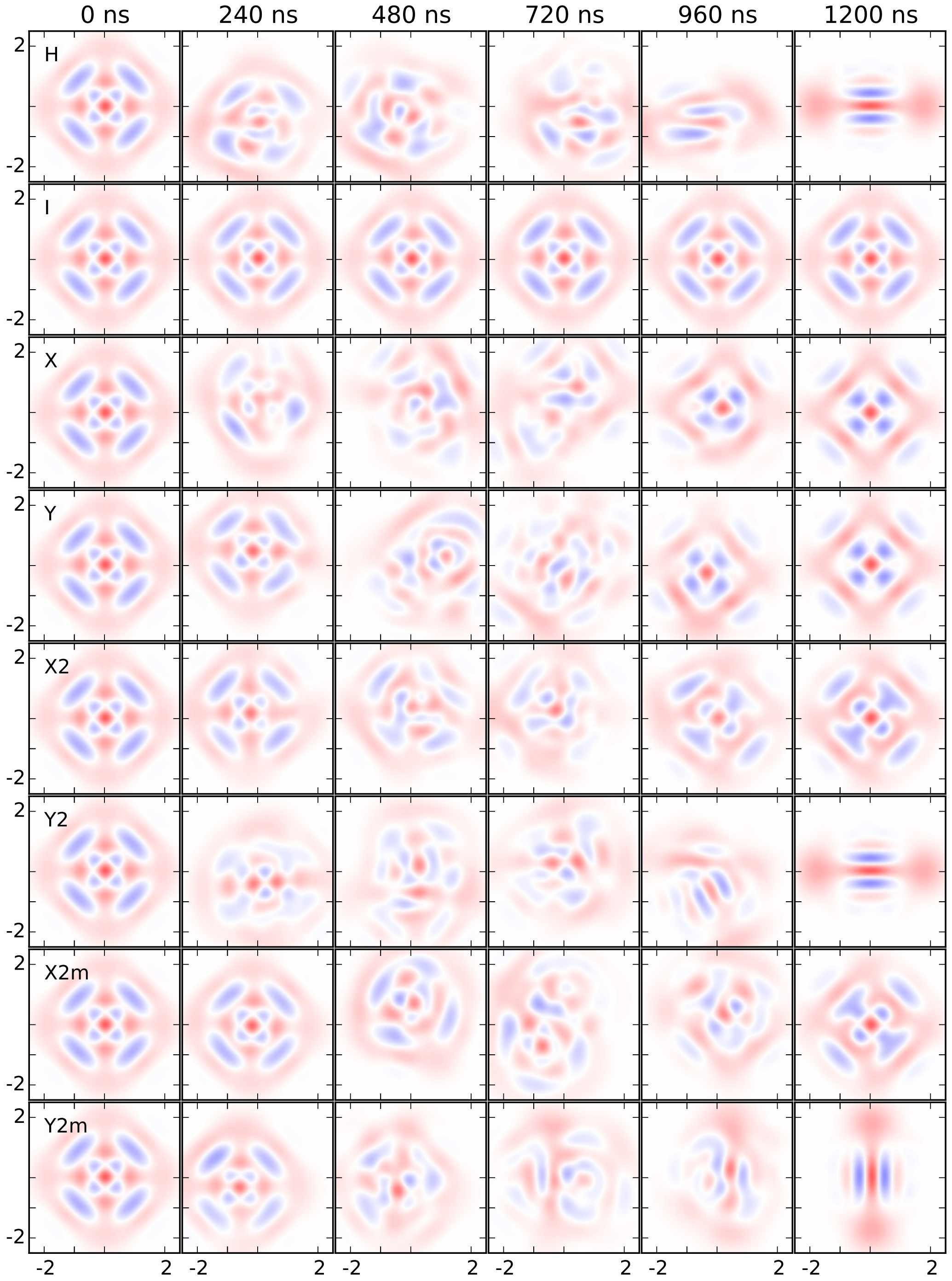

Supplementary Figure 10: **Wigner functions of state evolution**. Each row shows the evolution of the state $|+Z_L\rangle$ during a different operation. The snapshots at six different times correspond to the oscillator's Wigner function when tracing over the transmon degree of freedom.

SUPPLEMENTARY REFERENCES

[1] Byrd, R. H., Lu, P., Nocedal, J. & Zhu, C. A limited memory algorithm for bound constrained optimization. *SIAM Journal on Scientific Computing* **16**, 1190–1208 (1995).

[2] Najfeld, I. & Havel, T. F. Derivatives of the Matrix Exponential and Their Computation. *Advances in Applied Mathematics* **16**, 321–375 (1995).

[3] Motzoi, F., Gambetta, J. M., Merkel, S. T. & Wilhelm, F. K. Optimal control methods for rapidly time-varying Hamiltonians. *Phys. Rev. A* **84**, 022307 (2011).

[4] Heeres, R. W. *et al.* Cavity State Manipulation Using Photon-Number Selective Phase Gates. *Phys. Rev. Lett.* **115**, 137002 (2015).

[5] Epstein, J. M., Cross, A. W., Magesan, E. & Gambetta, J. M. Investigating the limits of randomized benchmarking protocols. *Phys. Rev. A* **89**, 062321 (2014).

[6] Chasseur, T. & Wilhelm, F. K. Complete randomized benchmarking protocol accounting for leakage errors. *Phys. Rev. A* **92**, 042333 (2015).

[7] Chen, Z. *et al.* Measuring and Suppressing Quantum State Leakage in a Superconducting Qubit. *Phys. Rev. Lett.* **116**, 020501 (2016).

[8] Wallman, J. J., Barnhill, M. & Emerson, J. Robust characterization of leakage errors. *New J. Phys.* **18**, 043021 (2016).

[9] Combes, J., Granade, C., Ferrie, C. & Flammia, S. T. Logical Randomized Benchmarking. *arXiv* 1702.03688v1.

[10] Egger, D. J. & Wilhelm, F. K. Adaptive Hybrid Optimal Quantum Control for Imprecisely Characterized Systems. *Phys. Rev. Lett.* **112**, 240503 (2014).

[11] Kelly, J. *et al.* Optimal Quantum Control Using Randomized Benchmarking. *Phys. Rev. Lett.* **112**, 240504 (2014).

[12] Kirchmair, G. *et al.* Observation of quantum state collapse and revival due to the single-photon kerr effect. *Nature* **495**, 205–209 (2013).
